# Neural response to reward uncertainty in adolescents with mood and anxiety symptoms

**DOI:** 10.1038/s41386-026-02412-3

**Published:** 2026-04-23

**Authors:** Qi Liu, Tram N. B. Nguyen, Russell H. Tobe, Emily R. Stern, Benjamin A. Ely, Vilma Gabbay

**Affiliations:** 1 Division of Brain Sciences, Changping Laboratory, Beijing, China.; 2 Department of Psychiatry and Behavioral Sciences, Albert Einstein College of Medicine, Bronx, NY, USA.; 3 Medical Scientist Training Program, Albert Einstein College of Medicine, Bronx, NY, USA.; 4 Nathan S. Kline Institute for Psychiatric Research, Orangeburg, NY, USA.; 5 Department of Psychiatry and Behavioral Sciences, University of Miami Miller School of Medicine, Miami, FL, USA.; 6 Center for the Developing Brain, Child Mind Institute, New York, NY, USA.; 7 Department of Psychiatry, New York University Grossman School of Medicine, New York, NY, USA.

## Abstract

Adolescence represents a critical neurodevelopmental period of high vulnerability to the onset of psychiatric conditions. Altered processing of uncertain reward outcomes likely contributes to this vulnerability, yet remains poorly understood. Addressing this knowledge gap, we sought to use the fMRI Reward Flanker Task, originally developed by our group, to examine neural responses to uncertain rewards and their clinical associations. To fully capture clinical correlates, we recruited adolescents with mood and anxiety symptoms ranging from low to high severity, including healthy controls (HC). Participants were 84 psychotropic-medication-free adolescents (15.3 ± 2.1 years; 62% female; 17 HC); all completed diagnostic and dimensional symptom assessments. Neuroimaging data were preprocessed using Human Connectome Project pipelines. Analyses examined participant-level neural responses to uncertain reward expectancy and attainment, adjusted for age, sex, and multiple comparisons. Across the whole sample, uncertain versus certain cues activated the default network and suppressed the fronto-parietal control network. Neural responses during expectancy to uncertain reward were intermediate between responses to certain reward and non-reward stimuli. Outcome attainment following uncertain cues activated stronger neural responses in reward and salience regions compared to reward cues. Anhedonia severity correlated with default network activation during uncertain outcome attainment. Anxiety severity correlated with blunted striatal responses during uncertain vs. certain non-reward expectancy. Exploratory group comparisons revealed that adolescents with mood and anxiety symptoms versus HC showed blunted striatal responses during uncertain versus non-reward expectancy and hyperactivation in visual and default network areas during attainment following uncertain cues. Together, these findings support the role of uncertain reward processing in adolescent mood and anxiety psychopathology.

## INTRODUCTION

Adolescence is a vulnerable period for developing psychiatric conditions, including depression, suicidality, anxiety, and substance use [1–3]. Before adolescence, the prevalence of depression is comparable between females and males [4]. Sex differences emerge in adolescence, with depression rates rising markedly among females to more than twice those observed in males, a disparity that persists into adulthood [4]. This pattern is thought to reflect divergent interactions among hormonal, psychosocial, and cognitive-affective factors between sexes [5]. The increased incidence of depression during adolescence coincides with rapid maturation and heightened plasticity of cortico-striatal circuits underlying reward function [6, 7], providing a developmental context in which such plasticity may also confer vulnerability to depression [8]. Importantly, reward function is a complex, multi-faceted construct involving reward expectancy, attainment, and valuation phases [9, 10]. Reduced neural activation during reward expectancy and attainment has been consistently documented in adults and adolescents with depression [11, 12]. Moreover, recent work from our laboratory has identified a distinct and inverted pattern of neural activity during reward expectancy compared to attainment that was diminished in youth with mood and anxiety symptoms [13, 14]. These findings underscore the importance of studying different phases and aspects of reward function.

Processing of uncertain reward represents another unique component of the reward function. Uncertainty is ubiquitous in daily life and frequently elicits distress [15]. Altered processing of reward uncertainty and subsequent responses may precipitate and perpetuate depression and anxiety [16, 17]. Notably, adolescence is characterized by heightened reward sensitivity [18] and greater tolerance for uncertainty [19], which together shape how rewards are processed under conditions of uncertainty. This pattern is consistent with dual systems models of adolescent neurodevelopment [6], which propose that reward circuitry matures earlier than cognitive control systems, promoting reward-driven exploration and greater engagement with uncertain outcomes. As such, altered responses to reward uncertainty during this critical developmental period may represent an important vulnerability for mood and anxiety psychopathology. Uncertainty also plays a central role in reinforcement learning, wherein discrepancies between expected and experienced rewards (i.e., reward prediction errors) are used to guide decision making and update behaviors in response to changing outcomes [15, 20, 21]. However, the neural mechanisms underlying reward uncertainty processing per se remain sparse, particularly in youth.

In prior studies, reward uncertainty has been implicitly examined using various probabilistic reward and reinforcement learning paradigms, which assess reward responsiveness as well as risk- and ambiguity-related decision making [22–24]. In contrast, our group sought to examine reward uncertainty as a distinct aspect of reward processing, as reflected in the incorporation of uncertain conditions in our original design of the Reward Flanker Task (RFT) [13]. Our task differs from these paradigms by employing predetermined reward or non-reward outcomes, excluding loss or punishment conditions. Additionally, responding to flanker stimuli requires active effort to obtain rewards, integrating motivational aspects that better approximate real-life reward acquisition contexts and thereby reducing potential confounds related to learning ability. Overall, this design enables more precise characterization of reward uncertainty processing during adolescence, when motivational systems undergo peak maturation [6]. Using the RFT, we previously examined neural responses to certain reward expectancy and to reward attainment following any cue type in a sample of 84 adolescents presenting with a wide range of mood and anxiety symptoms, including healthy controls (HC) [14]. In the current study, we leveraged the same dataset and performed a distinct set of analyses to focus on the neurocircuitry of reward uncertainty processing and its associations with clinical symptomatology.

Building upon previous findings from our laboratory and others, we hypothesized that: (1) Neural responses to uncertain reward would differ across expectancy and attainment phases and also be distinct from certain rewards or non-rewards; (2) Depression, anhedonia, and anxiety symptom severity would each correlate with distinct neural responses to uncertain reward. Additionally, we explored group differences in neural responses to uncertain reward between HC and adolescents with mood and anxiety symptoms.

## MATERIALS AND METHODS

### Participant recruitment and compensation

Adolescents were recruited from the New York metropolitan area through academic-affiliated clinics, physician referrals, and community advertisements. The Institutional Review Boards (IRBs) across all study sites approved all procedures prior to the study. Participants younger than 18 years provided signed assent, and a parent or legal guardian signed informed consent. Written informed consent was obtained from participants aged 18 and older.

Participants received $100–130 for completion of the neuroimaging portion of the study, depending on the year enrolled, and were informed they could earn up to an additional $24 based on their performance during the task. Additionally, participants were provided with up to $10 in compensation for travel expenses and up to $10 in light refreshments during the neuroimaging visit.

### Inclusion and exclusion criteria

The inclusion criteria for all participants were: (a) age between 12 and 20 years, (b) Tanner stage ≥ 4, (c) any biological sex, and (d) any race or ethnicity. All participants were required to be fluent in English. The study procedures were conducted in English to ensure consistency in task instructions and participant comprehension. Exclusion criteria included: (a) full-scale Intelligence Quotient (IQ) of 80 or below, assessed by the Kaufman Brief Intelligence Test (KBIT) [25], (b) current medical or neurological conditions, (c) active use of psychotropic or neuroactive medications, (d) magnetic resonance imaging (MRI) contraindications, (e) active suicidality, and (f) positive drug toxicology test or positive pregnancy test (female participants) on the day of scanning.

Information from clinician-based assessments (see below) was used to determine whether a participant belonged to the clinical or HC subgroup. Clinical participants were those with active psychiatric symptomatology. Additional exclusion criteria for the clinical subgroup included a current or past diagnosis of schizophrenia, autism spectrum disorder, or substance use disorders. HC participants had no psychiatric symptomatology and had never taken psychotropic medication.

### Clinical assessments

General psychiatric assessments were conducted using the Kiddie Schedule for Affective Disorders and Schizophrenia for School-Age Children–Present and Lifetime Version (K-SADS-PL) [26]. The K-SADS-PL is a semi-structured diagnostic interview with high reliability for identifying psychiatric symptoms and diagnoses based on criteria established in the Diagnostic and Statistical Manual of Mental Disorders (DSM) [27, 28]. In our protocol, all K-SADS-PL evaluations were administered to all participants by either a board-certified child and adolescent psychiatrist or a licensed clinical psychologist. In this study protocol, the K-SADS-PL instrument was based on DSM-IV criteria [27]. History of psychiatric disorders as determined by the K-SADS-PL was taken into account to determine study eligibility.

To capture symptoms dimensionally, we used various scales established in pediatric populations. Depression severity was measured through an interview with the child participant and, for enrollment age < 18, guardian by the clinician-rated Children’s Depression Rating Scale–Revised (CDRS-R) [29], which comprises 17 items and has a score range of 17 to 113. The inter-rater reliability analyses for the clinician-administered CDRS-R showed that the averaged intraclass correlation coefficient was 0.971 (95% CI: 0.887–0.996), indicating a high level of consistency in coding across raters [30]. Please see additional details in the Supplementary Methods and Supplementary Table S1. Anhedonia severity was assessed by the self-reported Temporal Experience of Pleasure Scale (TEPS) [31]. The TEPS has 18 items and a score range of 18 to 108. Anxiety severity was quantified using the self-reported Multidimensional Anxiety Scale for Children (MASC) [32], which contains 39 items with a score range of 0 to 117. Higher scores on the CDRS-R and MASC indicate greater symptom severity. Lower scores on the TEPS reflect more severe anhedonia.

### Neuroimaging data acquisition

We acquired all MRI data on a 3T Skyra scanner (Siemens, Germany) with a 16/4-channel head/neck coil. Our acquisition parameters were similar to those employed in the Human Connectome Project (HCP) LifeSpan protocols [33]. Specifically, high resolution (0.9 mm isotropic) anatomical images were acquired using the following sequences and parameters: (1) a T1-weighted MPRAGE sequence with TR/TI/TE = 2400/1000/2.06 ms, flip angle = 8°, FOV = 256 mm × 256 mm, 224 sagittal slices, and 0.9 mm slice thickness (no gaps); and (2) a T2-weighted SPACE sequence with TR/TE = 3200/566 ms, flip angle = 120°, FOV = 256 mm × 256 mm, 224 sagittal slices, and 0.9 mm slice thickness (no gaps).

Functional images were acquired at a 2.3 mm isotropic resolution using T2*-weighted gradient echo planar imaging (EPI) with the following parameters: TR/TE = 1000/31.4 ms, flip angle = 60°, FOV = 624 mm × 720 mm, and 5× multiband acceleration. For each run of the Reward Flanker Task (RFT, see below), 374 volumes were collected over 6 minutes and 14 seconds. Alternating LR/RL phase-encoding directions were used across 4 RFT runs. Additionally, for registration and distortion correction, a pair of spin-echo EPI fieldmaps with LR and RL phase encoding and matching parameters was acquired.

### Reward flanker task (RFT)

We have described the RFT in several previous publications [13, 14, 34–36]. Participants were first presented with a monetary reward cue (4–6 s), then a brief flanker stimulus (300 ms). If they correctly responded with button presses after identifying the target letter surrounded by four flanking letters during an allotted response interval (up to 1700 ms), they would earn the cued reward amount. The length of the response interval was calibrated for each participant based on performance during a pre-scan training session. Immediately after the response, a reward outcome slide (2 s) showed whether they responded correctly, incorrectly, or too slowly, in addition to the amount of money obtained or not obtained. Half of the trials presented certain cues, and the other half presented uncertain cues. The task employed a two-forced-choice response design.

The RFT included three levels of certain cues—high reward (“50¢”), low reward (“10¢”), no reward (“0¢”)—and one uncertain cue (“?”), which led to high (50¢), low (10¢) or no (0¢) reward with equal probability. Prior to the in-scanner RFT, participants completed a training session that explained the task, including the interpretation of the uncertain cues and possible outcome contingencies, and allowed the participants to practice the task in a simulated “mock” scanner under realistic conditions. Data from individuals’ training sessions were used to calibrate their in-scanner RFT response intervals, calculated as 150% of their mean response times in the final practice run (maximum of 1700 ms). The rationale for this response interval calibration is presented in our Supplementary Discussion. Unlike reinforcement learning paradigms, the RFT does not include a reward loss condition, thus limiting the possible involvement of negative valence outcomes. The RFT comprised a total of 120 trials, 30 per run, presented in pseudo-random order using a slow event-related design over four runs of approximately 6 minutes (374 s) each. Each run included 30 trials with an equal number of each trial type: cues were evenly divided between certain trials (5 × 0¢, 10¢, 50¢, 15 in total) and uncertain trials (15 ×?¢), and reward outcomes were equally proportioned following certain cues (5 × 0¢, 10¢, 50¢) and uncertain cues (5 × 0¢, 10¢, 50¢). Therefore, across the entire experiment, each participant completed a total of 60 certain-cue trials and 60 uncertain-cue trials. The pseudorandom trial order for each run was limited to a maximum of four consecutive certain or uncertain conditions to avoid habituation. At the end of each run, participants were informed of the total monetary amount they had earned. Supplementary Fig. S1 depicts example RFT trials of three possible correct feedback (50¢, 10¢, 0¢) following uncertain cues.

### Imaging data pre-processing

Preprocessing of MRI data used HCP minimal preprocessing pipelines version 3.2 [37]. These incorporate gradient non-linearity and fieldmap-based distortion correction for echo planar imaging (EPI) sequences, as well as realignment and normalization to the Montreal Neurological Institute (MNI) standard space and 32k CIFTI grayordinate templates. Two further advanced preprocessing techniques were applied: ICA-FIX noise removal and multimodal surface matching alignment (MSMAll). ICA-FIX utilizes independent component analysis (ICA) alongside an automated classifier to detect and remove structured noise from fMRI data [38, 39]. In our protocol, the multi-run ICA-FIX approach [40] was applied to the combined RFT scans and an additional 10-min (600-frame) resting-state fMRI scan acquired in the same session before the RFT. This step yielded components labeled as “signal,” “noise,” or “unknown”. All “signal” and “unknown” components were manually refined by trained neuroimagers in our group and reclassified as “noise” if necessary. The unique variance of “noise” components was then removed from the concatenated timeseries, while “signal” and “unknown” components were preserved. As in our prior RFT study [14], runs were defined as exhibiting excessive motion if >3% of frames had relative frame-to-frame root mean squared (RMS) displacement exceeding 1 mm, as estimated during the fMRIVolume step of the HCP pipelines. Runs exhibiting excessive motion were excluded from ICA-FIX and subsequent analyses, and participants were excluded if more than one RFT run exhibited excessive motion. Following ICA-FIX, MSMAll was employed to improve inter-participant cortical alignment. This technique leverages cortical myelination and fMRI-derived retinotopic and network properties to increase precision in mapping corresponding cortical regions, even across participants with substantial individual differences in cortical folding patterns [41, 42].

### Functional MRI analysis

#### Behavioral data analyses.

Task performance metrics, including accuracy and reaction time, were examined across the sample. Particularly, the mean response accuracy and reaction time to flanker stimuli following uncertain cues were compared to those following certain cues. Reaction time was analyzed only from correct trials. Additionally, whole-sample associations between symptom severity and task performance were determined using Spearman correlations.

#### Participant-level modeling.

We analyzed participant-level RFT data using Statistical Parametric Mapping (SPM) version 12 (Welcome Trust Centre for Neuroimaging, London, UK) within the MATLAB 2018b (The MathWorks, Inc.) environment. Following minimal spatial smoothing (4 mm FWHM) in CIFTI space, preprocessed fMRI data were converted to pseudo-NIFTI format using Connectome Workbench version 3.2.7. [37] to ensure compatibility with SPM. We modeled a total of eleven task-related regressors: four corresponding to cue conditions (0¢, 10¢, 50¢,?), six for correct feedback (0¢, 10¢, 50¢ outcomes, differentiated by certain or uncertain cues), and one for error feedback (incorrect or too-slow outcomes, when applicable). The jittered cue presentation timing (4–6 s in duration) with feedback following immediately post-response caused variable cue-feedback lag due to trial-by-trial response time and eliminated significant collinearity in general linear models. Subsequently, we convolved each regressor with a canonical hemodynamic response function within the general linear model (GLM) framework. To ensure adequate data, we excluded RFT runs lacking any trials across the 6 correct feedback regressors. Participants with fewer than three usable RFT runs were excluded from group-level analyses. Prior to group analyses, we converted all participant-level contrast maps back to CIFTI space.

To examine neural processing of uncertainty during expectancy and attainment phases, we employed the following primary participant-level contrasts. *Uncertain Outcome Expectancy* was defined as the difference in neural activation during uncertain cues (?) vs. certain cues (0¢ + 10¢ + 50¢). *Uncertain Outcome Attainment* is defined as the difference in neural activation when receiving any feedback (0¢ + 10¢ + 50¢) to uncertain cues compared to feedback to certain cues. To further parse the effect of uncertainty, we separately examined neural activation differences during reward and non-reward conditions using the following contrasts. *Uncertain Reward Expectancy*: uncertain (?) vs. certain reward (10¢ + 50¢) cues. *Uncertain Non-Reward Expectancy*: uncertain (?) vs. certain non-reward (0¢) cues. *Uncertain Reward Attainment*: correct reward feedback (10¢ + 50¢) following uncertain vs. certain reward cues. *Uncertain Non-Reward Attainment*: correct non-reward feedback (0¢) following uncertain vs. certain non-reward cues. High reward (50¢) and low reward (10¢) conditions were combined in this study, as the effects of different reward values were found to be largely similar to the combined reward effect during both the expectancy and attainment phases [14].

#### Group-level analyses.

To test our primary hypotheses, we examined mean activation across all participants and associations with clinical symptom scales in the full sample. Correlation analyses were performed to relate neural activities across contrasts with depression (CDRS-R), anhedonia (TEPS), and anxiety (MASC) severity. In secondary analyses, we conducted group comparisons to explore activation differences between mood and anxiety vs. HC subgroups. All analyses included sex and age as covariates of no interest.

Group-level analyses were performed in FSL PALM [43] using Threshold-Free Cluster Enhancement (TFCE) [44] and permutation-based non-parametric statistics to control for the family-wise error (FWE) rate. Due to the spatial dependence of TFCE and the discrete representation of major brain structures in CIFTI space, analyses were performed separately for cortical surfaces and for the bilateral subcortical volume, then merged. Results for the main analyses were considered significant at the two-tailed *p*_*TFCE-FWE*_ < 0.05 level across the whole brain. Information on significant clusters was reported based on the *ciftify_statclust_report* function, as implemented in Ciftify [45]. No additional multiple comparisons corrections were applied across group-level models. Group-level data are available upon request.

## RESULTS

### Demographic and clinical characteristics of study participants

The full study sample comprised 17 HC, 59 adolescents with mood and anxiety symptoms, and 8 adolescents with externalizing disorders (e.g., attention-deficit/hyperactivity disorder or oppositional defiant disorder) who endorsed no mood or anxiety symptoms. Table 1 details the demographic and clinical characteristics of the 84 study participants, representing the identical sample analyzed in our prior manuscript [14].

The full sample had a mean age of 15.3 ± 2.14 years and was 62% female. There was a significant difference in the female/male ratio observed between the mood and anxiety group (41 females vs. 18 males) and the HC group (6 females vs. 11 males; *χ*^2^ = 6.54, *p* = 0.01). However, no significant difference was found in age between the two groups (15.25 ± 2.14 vs. 15.65 ± 2.47; both ranging from 12 to 20 years; *t* = −0.75, *p* = 0.46).

Across the full sample, the mean ± SD scores were 31.9 ± 14.6 for the CDRS-R, 41.5 ± 17.1 for the MASC, and 81.1 ± 13.8 for the TEPS. Depression was significantly correlated with anxiety (*r* = 0.52; *p* < 0.001) and anhedonia (*r* = −0.47; *p* < 0.001), but the correlation between anxiety and anhedonia was not significant (*r* = −0.05; *p* = 0.68). Supplementary Table S2 presents the correlation matrix across symptoms.

### Uncertain reward task behavioral analysis

In the full sample, participants made significantly slower responses to flanker stimuli following uncertain cues (697.88 ± 118.63 ms) relative to certain cues (685.73 ± 111.66 ms) (*t*(83) = 3.03, *p* = 0.003). There was no significant relationship between reaction time and depression, anhedonia, and anxiety in the full sample.

Accuracy following uncertain cues (87.26 ± 9.36%) and certain cues (85.99% ± 9.89%) did not differ significantly (*t*(83) = 1.91, *p* = 0.06). More severe consummatory anhedonia (lower TEPS-CP scores) was significantly correlated with lower accuracy following both uncertain cues (*ρ* = 0.24, *p*_*unc*_ = 0.049) and certain cues (*ρ* = 0.27, *p*_*unc*_ = 0.02) prior to multiple comparison correction, though these correlations did not remain significant after correction. No other significant associations between accuracy and clinical symptoms were observed in the full sample.

### Neural activation to uncertainty during expectancy and attainment

**Fig. 1:**
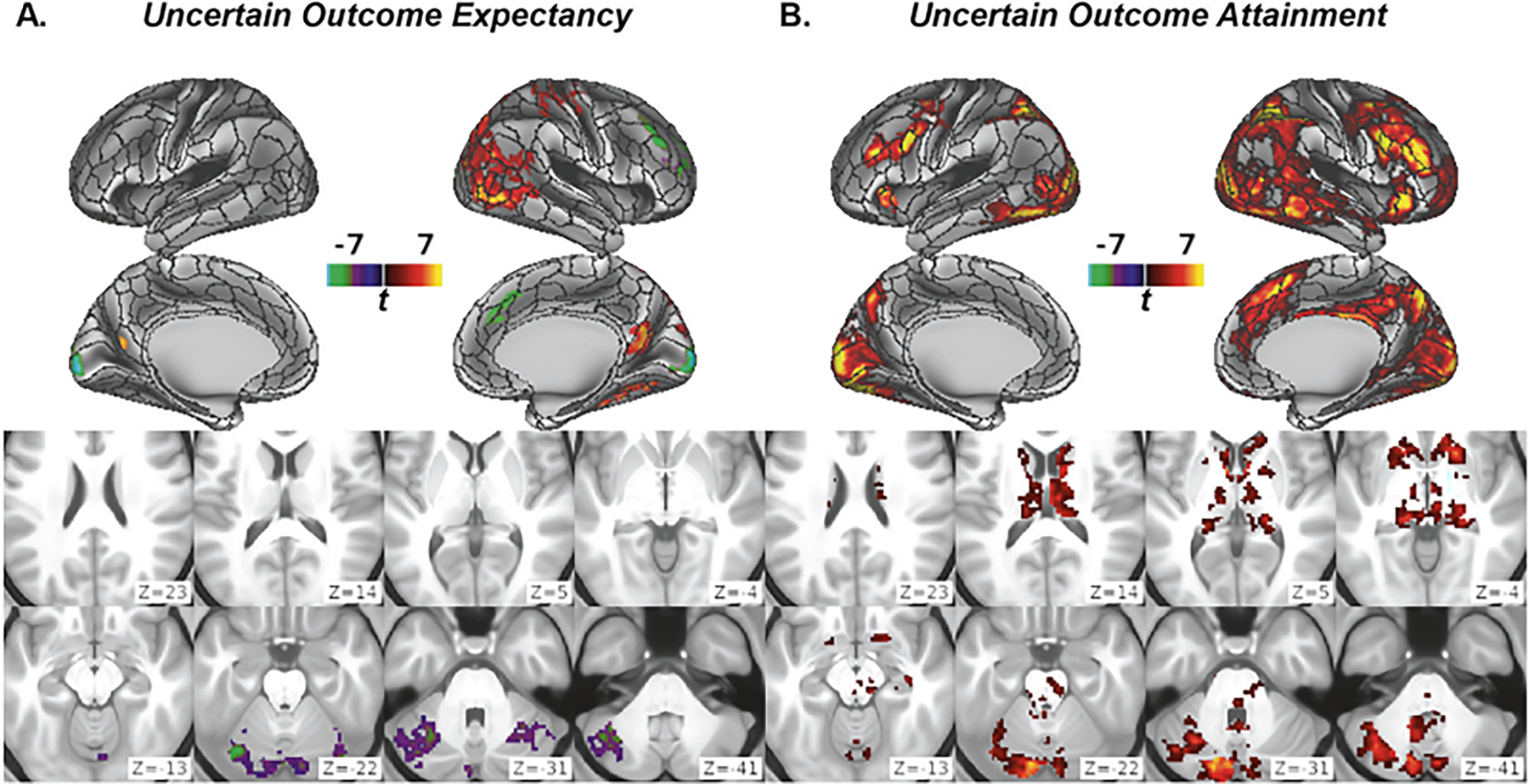
Neural activation during. **A** *Uncertain Outcome Expectancy* (uncertain vs. certain cues) and **B** *Uncertain Outcome Attainment* (feedback after uncertain cues vs. feedback after certain cues): All participants. Group-level activation maps (whole sample) are displayed at the two-tailed *p*_*TFCE-FWE*_ < 0.05 threshold. Sulcal depth from the HCP 1200-participant dataset is shown in the background. Black surface contours denote regions from the HCP MMP Atlas, Glasser et al. [41]. Subcortical results are displayed in neurological convention.

Maps showing significant (two-tailed *p*_*TFCE-FWE*_ < 0.05) neural activation patterns for uncertainty during expectancy and attainment are displayed in Fig. 1. Activation maps examining uncertainty separately for reward and non-reward conditions are presented in Supplementary Fig. S2. To avoid ambiguity due to overlapping spatially extended activation clusters, cortical sub-peaks reported in all tables were defined as the upper and lower 5% of activation magnitudes for significant vertices within each contrast.

#### Uncertain outcome expectancy.

Relative to certain cues, uncertain cues elicited stronger activation in the bilateral posterior cingulate cortex (PCC), right sensorimotor cortex, superior parietal lobule (SPL), lateral occipital complex (LOC), and fusiform gyrus (Fig. 1A and Table 2). These regions span the default mode (DMN), somatomotor, and visual networks. Conversely, certain cues evoked stronger activation in the right inferior frontal gyrus (IFG), dorsal anterior cingulate cortex (dACC), occipital pole, and cerebellum, which are part of the fronto-parietal and visual networks.

Separate analyses of reward and non-reward conditions revealed that neural responses to uncertainty were stronger for non-rewards than rewards during expectancy (uncertain vs. certain cues). Specifically, the fronto-striatal reward and salience networks exhibited reduced activation when processing uncertain cues relative to certain reward cues (*Uncertain Reward Expectancy*; Supplementary Fig. S2A). In contrast, these networks showed greater activation in response to uncertain cues compared to certain non-reward cues (*Uncertain Non-Reward Expectancy*; Supplementary Fig. S2B), such that activation in certain (10¢ and 50¢) reward > uncertain (?) reward > non-reward (0¢). Additionally, *Uncertain Reward Expectancy* evoked less activation across the occipital cortex, somatomotor areas, SPL, and inferior frontal cortex (IPC), as well as the thalamus, cerebellum, and brain stem. Conversely, these regions, along with the amygdala, were more strongly activated during *Uncertain Non-Reward Expectancy*. A list of significant clusters for these two contrasts is provided in Supplementary Table S3.

#### Uncertain outcome attainment.

This contrast examined neural activation during feedback following uncertain vs. certain cues. Stronger activation was primarily observed in reward and salience regions, including the bilateral anterior insula, IFG, LOC, striatum, thalamus, cerebellum, as well as the right dACC and PCC (Fig. 1B and Table 2).

There was a robust differentiation in neural responses to outcome attainment following uncertain versus certain reward cues (*Uncertain Reward Attainment*; Supplementary Fig. S2C). Particularly, *Uncertain Reward Attainment* activated the fronto-striatal reward network, visual cortex, IFG, SPL, LOC, right anterior insula, dACC, PCC, brain stem, and cerebellum. In contrast, neural activation during uncertain versus non-reward feedback attainment (*Uncertain Non-Reward Attainment*; Supplementary Fig. S2D) was limited to the bilateral occipital lobe, intraparietal sulcus, and temporal cortex. Significant clusters across these two contrasts are listed in Supplementary Table S4.

### Dimensional associations with mood and anxiety symptoms

**Fig. 2:**
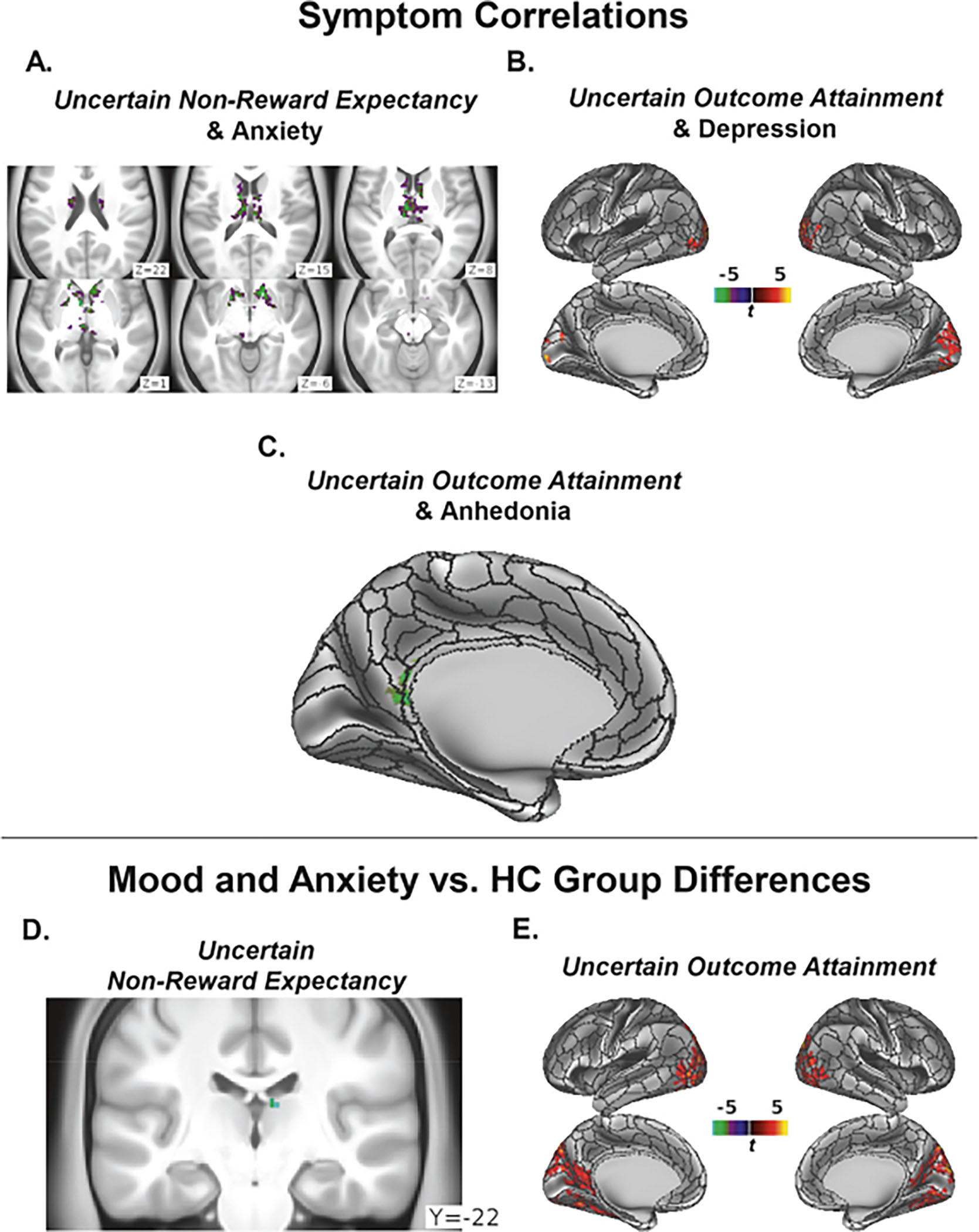
Neural processing of reward uncertainty: symptom correlates across the full sample and exploratory group differences. In the whole sample, correlations between **A** *Uncertain Non-Reward Expectancy* activation and anxiety severity (MASC), **B** *Uncertain Outcome Attainment* activation and depression severity (CDRS-R), and **C** *Uncertain Outcome Attainment* activation and anhedonia severity (TEPS) are depicted. As lower scores on the TEPS indicate more severe anhedonia, there was a positive correlation between neural activation and anhedonia levels. Group differences in neural activation between the mood and anxiety subgroup and healthy controls (HC) are presented for: **D** *Uncertain Non-Reward Expectancy*, and **E** *Uncertain Outcome Attainment*. Group-level activation maps are displayed at the two-tailed *p*_*TFCE-FWE*_ < 0.05 threshold. Sulcal depth from the HCP 1200-participant dataset is shown in the background. Black surface contours denote regions from the HCP MMP Atlas Glasser et al., [41]. Subcortical results are displayed in neurological convention.

In the full sample, anxiety severity was negatively correlated with neural activation during *Uncertain Non-Reward Expectancy* across bilateral striatum and thalamus (Fig. 2A). Neural responses during *Uncertain Outcome Attainment* were associated with both depression (Fig. 2B) and anhedonia severity (Fig. 2C). Specifically, CDRS-R scores were positively correlated with LOC activation, whereas total TEPS scores were negatively correlated with activation in the LOC and retrosplenial cortex. Given that higher CDRS-R indicates greater depression levels while lower TEPS indicates greater anhedonia levels, greater symptom severity was associated with stronger activation in these regions. Details are further provided in Table 3.

### Exploratory group comparisons

Significant group differences in neural activation were observed during *Uncertain Non-Reward Expectancy*, with the mood and anxiety subgroup exhibiting blunted activation in the right caudate relative to HC (Fig. 2D and Table 3). During *Uncertain Outcome Attainment*, the bilateral posterior visual cortex, covering the occipital cortex, cuneus, lingual and fusiform gyrus, was more strongly activated in adolescents with mood and anxiety symptoms (Fig. 2E and Table 3).

## DISCUSSION

This study used the fMRI Reward Flanker Task in a transdiagnostic sample of adolescents to investigate uncertainty in reward function. As hypothesized, reward uncertainty elicited distinct neural responses across expectancy and attainment phases. Neural responses to uncertainty also differed between rewards and non-rewards. Clinically, depression, anhedonia, and anxiety levels correlated with divergent profiles of neural responses to uncertain reward. Beyond our previous work using the same dataset [14], the current study substantially advances the literature by: (a) explicitly modeling neural responses to uncertain versus certain conditions during reward expectancy and attainment, (b) differentiating neural activation across these phases of reward uncertainty processing; and (c) linking distinct aspects of reward uncertainty processing to depression, anhedonia, and anxiety symptomatology in youth. Together, these findings provide novel insights into the neurocircuitry underlying reward uncertainty processing and its contribution to these highly prevalent clinical conditions.

Behaviorally, responses to flanker stimuli following uncertain cues took significantly longer than following certain cues, though accuracy did not differ between uncertain and certain cue conditions. To our knowledge, this is the first report to directly compare reaction time and accuracy between reward uncertainty and certainty processing among adolescents. These findings suggest that the concept of reward uncertainty requires increased processing time relative to certainty, potentially to accommodate higher neural demands.

In our neuroimaging analysis, reward uncertainty during both expectancy and attainment engaged the dorsal attention, fronto-parietal, somatomotor, and visual networks, likely to divert attention toward cognitive and sensory processing of cue stimuli [46, 47]. Compared with our prior findings on certain reward processing [14], reward uncertainty recruited more restricted neural networks with distinct features. Uncertain cues enhanced DMN activity and, while dampening fronto-parietal network activity relative to certain cues, suggesting a discrete mixed state of heightened self-referential focus and suppressed executive control [46]. In addition to evoking these networks, certain cues also activated the salience network [14]. A notable feature of reward uncertainty processing uncovered in this study was the consistent activation of expectancy-related networks in the attainment phase. This contrasts with previously reported certain reward processing, where expectancy-phase networks showed reduced activation during attainment [14]. Notably, striatal responses to uncertainty were minimal during expectancy but heightened during attainment, which is the opposite of the pattern we observed in certain reward processing [14]. Thus, uncertain reward may prompt reflection on past experiences and reduce goal-directed preparation during expectancy. Consistent with our findings in youth, decision-making under uncertainty in adults engaged the visuomotor pathway along with frontal and cingulate control regions to integrate sensory information with prior experiences [48]. Similar neural representations of reward uncertainty have also been documented in non-human primates [49]. In our data, outcomes following uncertainty appear highly salient and inherently rewarding to adolescents regardless of monetary value, potentially facilitating memory formation and prediction adjustment [49]. This result is consistent with adolescent development patterns, with peak reward sensitivity emerging as cognitive control systems approach adult levels [6]. Such a trajectory likely serves an adaptive role in reinforcement learning [50, 51], enabling adolescents to progressively refine their behavior to balance between risks and rewards.

Our study used valence-specific contrasts to examine how uncertainty modifies neural processing of cues and outcomes relative to known reward and non-reward values. Neural responses to uncertain cues across visual, fronto-parietal, dorsal attention, and reward networks were stronger than to non-reward cues, yet weaker than to reward cues. This indicates that processing reward uncertainty engages attentional and cognitive systems at a level intermediate between certain non-rewards and rewards. The additional engagement of the DMN and salience network during uncertain versus non-reward expectancy may be important for enhanced coordination between internal focus and external stimulus processing. Resolving uncertainty engages large-scale networks more robustly for rewards than non-rewards, possibly indicating greater importance for updating priors and reshaping perceptions for future decision-making [48].

With respect to clinical associations, in the full sample, blunted activity in the bilateral ventral striatum, a key subcortical reward region [52, 53], during uncertain vs. non-reward cues negatively correlated with more severe anxiety. Depression and anhedonia severity were linked to lateral occipital hyperactivity during uncertain outcome attainment, with anhedonia also associated with retrosplenial hyperactivity. These findings are in line with our prior work showing neural alterations in anxiety during reward expectancy and in depression during reward attainment [14]. Our exploratory analyses further revealed that adolescents with mood and anxiety symptoms showed suppressed right caudate activity when processing uncertain vs. non-reward cues, as well as heightened activity in the default and visual networks during resolution of uncertain outcomes, relative to their healthy counterparts. Taken together, our findings support the notion that altered processing of reward uncertainty is a transdiagnostic feature of both depression and anxiety [16, 54], but that distinct phases of this process are differentially implicated in each condition.

Prior work using probabilistic reward and gambling tasks indicates that adolescence is marked by developmentally distinct patterns of decision making under uncertainty. Specifically, relative to children and adults, adolescents exhibit greater tolerance for uncertainty [19, 55]. Furthermore, in prior studies using probabilistic reward tasks, adolescents at high risk for MDD showed blunted response bias toward a more frequently rewarded stimulus relative to those at low risk [56]. In addition, adolescents with co-occurring depression and substance use disorders exhibited improved reward responsiveness following short-term residential treatment [57]. These data support a role for altered reward learning under uncertainty in mood vulnerability during this developmental period. Our current study, employing the Reward Flanker Task, extends this literature by specifying neural responses to reward uncertainty across distinct phases of expectancy and attainment. Although depression, anhedonia, and anxiety frequently co-occur among adolescents, our results reveal divergent neural mechanisms underlying uncertainty processing in relation to each of these symptom dimensions.

Overall, our documentation of varied neural markers across symptoms highlights the potential of utilizing neuroimaging for improving therapeutic precision. Given reports on lateral occipital hyperactivation during both reward anticipation and attainment following psychotherapy [58], uncertain reward processing deficits identified in this study suggest that the underlying neural circuits may represent a promising target for future intervention development in mood and anxiety-related symptoms in adolescents. In addition, activation in the retrosplenial cortex, which is part of the DMN [59], was positively correlated with anhedonia severity. It is possible that as uncertain outcomes become clear, youth experiencing more severe anhedonia are more prone to rumination, a process associated with altered DMN activity [60]. These findings also support our prior reports on anhedonia as a neurobiologically distinct construct from depression [61–63]. Further, among more anxious youth, the certainty of non-reward may be more positively valued than uncertain reward, as reflected by striatal responses. This finding adds nuance to the well-documented maladaptive responses of reward regions to uncertainty in anxiety [64]. Therefore, these findings suggest that interventions should target how adolescents appraise reward uncertainty, including reducing pessimistic biases in anxiety and limiting attentional vigilance or internalization of uncertainty in depression and anhedonia.

There are several caveats to consider when interpreting the present findings. First, although our sample comprised a relatively large number of psychotropic-medication-free adolescents, participants predominantly reported mood and anxiety symptoms. This clinical diversity may constrain the ability to map findings onto single disorders but improves generalizability. The variable symptom severity within our sample also allowed us to capture a wide spectrum of reward functions, providing sufficient power to detect effects in correlation analyses. *Post hoc* sensitivity analyses, performed using G*Power v3.1 [65], indicated that we achieved 80% power to detect correlations as small as |*ρ* | = 0.30 in our full sample, corresponding to moderate or larger effects. Across the mood and anxiety group (*n* = 59) and healthy controls (*n* = 17), *post hoc* sensitivity analysis indicated 80% power to detect effects of |d | ≥ 0.78, corresponding to moderate-to-large effect sizes. Therefore, group comparisons were considered exploratory. Second, the relatively broad age range across adolescence (12–20 years old) should be noted. Importantly, all participants had reached the later stages of puberty (Tanner stage ≥ 4; all female participants reached menarche), thus minimizing hormonal influences on development. Future studies should incorporate hormone levels and menstrual cycle phase to disentangle the effects of cyclic hormonal fluctuations on reward processing in female adolescents. Although we controlled for age and sex, our sample size and sex imbalance limited the power to test sex-specific effects. Accordingly, larger studies with balanced sex representation are needed to evaluate sex differences in neural mechanisms linking reward uncertainty processing to mood and anxiety symptoms. Third, the cross-sectional nature of our analyses limited inferences about how observed differences in neural dysfunction in processing uncertain reward may predict future clinical outcomes. Follow-up longitudinal studies with larger cohorts are needed to clarify the neural underpinnings of reward uncertainty in relation to symptom trajectories. Finally, future studies should include direct assessments of Intolerance of Uncertainty (IU) and punishment uncertainty processing to disentangle the unique contributions of reward-specific uncertainty alterations from broader IU-related vulnerabilities in mood and anxiety symptoms [66].

In conclusion, our findings illustrate the impact of uncertainty on both reward expectancy and attainment processing. Uncertain reward expectancy may prompt a mental state characterized by internally directed reflection, heightened sensory monitoring, and suppressed cognitive control. Reward uncertainty may influence outcome processing, associated with widespread activation across reward, salience, and internally and externally focused attention systems. Critically, uncertain reward processing impairments were linked to adolescent mood and anxiety symptoms via distinct neural profiles. As reward uncertainty is highly relevant to real-world settings, further investigations into its role may inform the development of practical, targeted interventions for high-risk populations.

## Supplementary Material

Supplementary Material

**Supplementary information** The online version contains supplementary material available at https://doi.org/10.1038/s41386-026-02412-3.

## Figures and Tables

**Table 1.** Demographic and clinical characteristics of study participants.

***Demographics—Full Sample (N = 84)***			
Age (Years)	15.3 ± 2.14 (12–20)	Ethnicity (Caucasian/African American/Other)	39/28/17 (46.43/33.33/20.24)
Sex (F/M)	52/32 (61.9/38.1)		
Psychiatric group	67(79.76)	HC	17 (20.24)
***Clinical Symptom Measures—Full Sample (N = 84)***			
CDRS-R ^a^	31.93 ± 14.6 (17–78)	MASC ^b^	41.49 ± 17.14 (2–87)
TEPS ^c^	81.11 ± 13.82 (40–105)	TEPS-AP ^c^	46.89 ± 8.05 (20–60)
TEPS-CP ^c^	34.22 ± 7.93 (11–48)		
***DSM Diagnoses (Current/Past)—Psychiatric Group (n = 67)***			
MDD	30/5 (44.78/7.46)	Other Mood Disorder	3/2 (4.48/2.99)
Dysthymia	4/0 (5.97/0)	OCD	2/1 (2.99/1.49)
DDNOS	3/0 (4.48/0)	ODD	7/0 (10.45/0)
Bipolar Disorder II	3/0 (4.48/0)	ADHD	21/1 (31.34/1.49)
Anxiety	42/1 (62.69/1.49)	Other	5/2 (7.46/2.99)
Med-naïve/Med-free	56/11 (83.58/16.42)		
***Demographics—Mood and Anxiety Subgroup (n = 59)***			
Age (Years)	15.25 ± 2.14 (12–20)	Ethnicity (Caucasian/ African American/Other)	30/16/13 (50.85/27.12/22.03)
Sex (F/M)	41/18 (69.49/30.51)		
***Clinical Symptom Measures—Mood and Anxiety Subgroup (n = 59)***			
CDRS-R ^a^	37.09 ± 14.55 (17–78)	MASC	44.80 ± 16.55 (11–87)
TEPS ^d^	79.62 ± 13.25 (40–102)	TEPS-AP ^d^	45.18 ± 8.10 (20–60)
TEPS-CP ^d^	33.18 ± 7.37 (14–48)		
***Demographics—HC Subgroup (n = 17)***			
Age (Years)	15.65 ± 2.47 (12–20)	Ethnicity (Caucasian/African American/Other)	6/7/4 (35.29/41.18/23.53)
Sex (F/M)	6/11(35.29/64.71)		
***Clinical Symptom Measures—HC Subgroup (n = 17)***			
CDRS-R	18.29 ± 1.53 (17–22)	MASC ^e^	26.43 ± 12.47 (2–49)
TEPS	85.94 ± 14.92 (53–105)	TEPS-AP	49.88 ± 7.00 (33–58)
TEPS-CP	36.06 ± 9.72 (11–48)		

Values reported as M ± SD (Range) or *n* (%), as appropriate. Diagnoses were based on the DSM-IV to maintain consistency across all participants over time. As participants could meet full or subthreshold criteria for more than one disorder, totals may not sum to 100%.

*ADHD* attention-deficit/hyperactivity disorder, *Anxiety* includes generalized anxiety, social anxiety, *phobia,* post-traumatic stress, panic disorders, and anxiety disorder not otherwise specified, *CDRS-R* Children’s Depression Rating Scale-Revised, *DDNOS* depressive disorder not otherwise specified; *HC* healthy controls with no history of psychiatric illness, MASC Multidimensional Anxiety Scale for Children, *MDD* major depressive disorder, *OCD* obsessive-compulsive disorder, *ODD* oppositional defiant disorder, *TEPS* Temporal Experience of Pleasure Scale, *TEPS-AP/-CP* TEPS, Anticipatory/Consummatory Pleasure subscales.

Data missing for:

a 1 participant.

b 4 participants.

c 12 participants.

d 9 participants.

e 3 participants.

**Table 2.** Neural Activation during Uncertain Outcome Expectancy and Uncertain Outcome Attainment.

*Uncertain Outcome Expectancy*					
	Peak *T*	Area (mm^2^)	Cluster Overlap: Desikan-Killiany Atlas (Desikan [67])	Cluster Overlap: HCP MMP Atlas (Glasser [41])	Cluster Overlap: 7-Network iFC Atlas (Yeo [68])
R	7.131	7332.965	inferiorparietal (33.5%)	PGi (8.8%)	Visual (50.8%)
R	3.837	1044.683	postcentral (57.3%)	2 (41.5%)	Somatomotor (80.9%)
R	6.168	752.2228	precuneus (94.1%)	POS1 (73.2%)	Default (77%)
R	6.608	701.9864	lingual (47.8%)	VMV2 (35.1%)	Visual (98.5%)
R	3.374	455.1017	precentral (70.2%)	4 (38.4%)	Somatomotor (100%)
R	4.118	342.9236	superiorparietal (100%)	VIP (56.3%)	Dorsal Attention (100%)
R	3.146	81.66182	precentral (100%)	6 d (71.6%)	Somatomotor (100%)
L	6.091	79.74442	precuneus (100%)	POS1 (100%)	Default (86.1%)
R	−8.236	1698.799	lateraloccipital (64.2%)	V1 (63.5%)	Visual (100%)
R	−6.338	1363.767	rostralmiddlefrontal (98.7%)	9-46 d (55%)	Frontoparietal (82.2%)
R	−6.433	737.8152	rostralmiddlefrontal (72.9%)	p9-46v (35.4%)	Frontoparietal (100%)
L	−9.871	604.4233	lateraloccipital (63.8%)	V1 (88.1%)	Visual (100%)
R	−6.175	390.1869	superiorfrontal (95.9%)	a32p (39.9%)	Frontoparietal (77.7%)
	Peak *T*	Volume (mm^3^)	Peak Coordinates (MNI X,Y,Z)	Brain Region	
	−5.85	13952	(−44, −64, −30)	left cerebellum	
	−5.65		(−32, −68, −20)		
	−5.25		(−44, −74, −28)		
	−5.24	3040	(30, −60, −28)	right cerebellum	
	−4.48		(42, −74, −24)		
	−4.19		(34, −52, −30)		
	−3.23	40	(34, −38, −28)	right cerebellum	
*Uncertain Outcome Attainment* ^a^					
	Peak *T*	Area (mm^2^)	Cluster Overlap: Desikan-Killiany Atlas (Desikan [67])	Cluster Overlap: HCP MMP Atlas (Glasser [41])	Cluster Overlap: 7-Network iFC Atlas (Yeo [68])
L	9.311	3447.42	lateraloccipital (49.1%)	V1 (16.3%)	Visual (72.5%)
R	10.251	2353.962	inferiorparietal (58.4%)	IP1 (26.4%)	Frontoparietal (56.7%)
L	9.112	2257.795	fusiform (34.3%)	V4 (22.3%)	Visual (80.4%)
R	9.617	1618.813	rostral middle frontal (65.7%)	p9-46v (36.7%)	Frontoparietal (76.1%)
R	7.97	1608.049	lateraloccipital (83.2%)	V4 (32.1%)	Visual (100%)
R	7.851	641.2761	lateraloccipital (59.5%)	V4 (39.3%)	Visual (100%)
R	7.737	496.4383	precuneus (82.6%)	POS2 (86.0%)	Frontoparietal (59.6%)
R	7.757	468.7598	inferiortemporal (69%)	TE1p (93.4%)	Frontoparietal (75.9%)
R	7.904	419.3742	insula (63.2%)	AVI (69.3%)	Ventral Attention (57.9%)
L	7.623	377.0561	precentral (53.3%)	IFJp (67.5%)	Frontoparietal (55.6%)
	Peak *T*	Volume (mm^3^)	Peak Coordinates (MNI X,Y,Z)	Brain Region	
	7.42	28952	(20, 12, −6)	bilateral thalamus, caudate, putamen	
	6.03		(8, −14, 8)		
	5.66		(−6, 2, 4)		
	6.92	24656	(−10, −80, −24)	left cerebellum	
	6.84		(−12, −76, −36)		
	6.79		(−4, −80, −34)		
	5.33	4664	(−14, −44, −44)	left cerebellum	
	5.2		(−8, −58, −38)		
	4.79		(−6, −50, −46)		
	4.91	88	(30, −72, −46)	right cerebellum	
	4.74		(34, −72, −50)		
	3.68	56	(−14, −22, −28)	brain stem	
	3.65		(−14, −18, −32)		

a 5% threshold for positive activation t-value is 5.171.

Only the largest 10 clusters and the largest parcellation labels are listed here. To characterize sub-peak locations, this table includes information on all subcortical clusters larger than 40 mm3 and the 10 largest cortical sub-peaks. Cortical findings include overlap with three widely used cortical atlases—the Desikan-Killiany atlas implemented in Freesurfer (Desikan et al. [67]), HCP multimodal parcellation (MMP) atlas Glasser et al. [41], and 7-network intrinsic functional connectivity (iFC) atlas (Yeo et al. [68]).

**Table 3.** Clinical correlates of neural processing of uncertain reward.

*Uncertain Non-Reward Expectancy* & Anxiety (MASC)				
	Peak *T*	Volume (mm^3^)	Peak Coordinates (MNI X,Y,Z)	Brain Region
	−4.91	11600	(−6, 6, 0)	bilateral thalamus, caudate, putamen, accumbens,	
	−4.62		(−18, 8, −2)		
	−4.46		(−6, −16, 10)		
	−4.01	136	(14, −74, −50)	right cerebellum	
	−3.76		(16, −74, −44)		
	−4.87	88	(32, −56, −20)	right cerebellum	
	−4.45	48	(−2, −50, −32)	left cerebellum	
*Uncertain Outcome Attainment* & Depression (CDRS-R)					
	Peak *T*	Area (mm^2^)	Cluster Overlap: Desikan-Killiany Atlas (Desikan [67])	Cluster Overlap: HCP MMP Atlas (Glasser [41]	Cluster Overlap: 7-Network iFC Atlas (Yeo [68])
L	4.714	1968.797	lateraloccipital (94.6%)	V4 (29.2%)	Visual (100%)
R	4.785	5231.286	lateraloccipital (42.6%)	V2 (25.9%)	Visual (100%)
L	3.848	220.092	cuneus (92.1%)	V2 (81.5%)	Visual (100%)
L	5.26	179.915	lateraloccipital (50.0%)	V1 (100.0%)	Visual (100%)
*Uncertain Outcome Attainment* & Anhedonia (TEPS)					
	Peak *T*	Area (mm^2^)	Cluster Overlap: Desikan-Killiany Atlas (Desikan [67])	Cluster Overlap: HCP MMP Atlas (Glasser [41]	Cluster Overlap: 7-Network iFC Atlas (Yeo [68])
L	−4.054	144.394	isthmuscingulate (70.5%)	POS1 (46.4%)	Default (100%)
L	−4.815	126.598	lateraloccipital (100%)	V3 (85.1%)	Visual (100%)
*Uncertain Non-Reward Expectancy* (HC > Mood & Anxiety)					
	Peak *T*	Volume (mm^3^)	Peak Coordinates (MNI X,Y,Z)	Brain Region	
	4.86	48	(10, −22, 14)	right caudate	
*Uncertain Outcome Attainment* (Mood & Anxiety > HC)					
	Peak *T*	Area (mm^2^)	Cluster Overlap: Desikan-Killiany Atlas (Desikan [67])	Cluster Overlap: HCP MMP Atlas (Glasser [41]	Cluster Overlap: 7-Network iFC Atlas (Yeo [68])
L	5.16	7130.991	lateraloccipital (37.4%)	V2 (13%)	Visual (88.2%)
R	5.762	4800.776	lateraloccipital (27.6%)	V2 (20.2%)	Visual (97.2%)
L	4.181	2307.574	fusiform (47.7%)	V2 (21.3%)	Visual (89.4%)
R	4.932	3763.948	lingual (40.1%)	V2 (18.6%)	Visual (97.3%)

Only parcellation labels with the largest overlap are listed here.

*CDRS-R* Children’s Depression Rating Scale-Revised, *HC* healthy controls, MASC Multidimensional Anxiety Scale for Children, Mood & Anxiety: group of adolescents with mood and anxiety symptoms, *TEPS* Temporal Experience of Pleasure Scale.

## Data Availability

Data from the current study are available from the corresponding author upon reasonable request.
