# Supplementary material for: Neural response to reward uncertainty in adolescents with mood and anxiety symptoms
Source: Neuropsychopharmacology. 2026 Apr 23;51(9):1623–33. doi: 10.1038/s41386-026-02412-3 (PMC13278348; doi:10.1038/s41386-026-02412-3)
Supplement: Supplementary file 1 — Supplementary Materials [file 41386_2026_2412_MOESM1_ESM.docx]

**SUPPLEMENTARY MATERIALS**

**Neural Response to Reward Uncertainty in Adolescents with Mood and Anxiety Symptoms**

Qi Liu, Tram N. B. Nguyen, Russell H. Tobe, Emily R. Stern,

Benjamin A. Ely, Vilma Gabbay

# Supplementary Methods

## **Inter-rater Reliability of Clinician-Administered Assessments**

Psychiatric diagnostic evaluations using the K-SADS-PL [1] were administered to all participants by clinical evaluators that were either a board-certified child and adolescent psychiatrist or a licensed clinical psychologist/social worker. Clinical evaluators participated in rigorous diagnostic measure-specific training including didactics, role play, and certification administrations observed by a study PI (VG or RHT). A weekly diagnostic consensus meeting run by the study PIs including clinician evaluators reviewed participant-level data, symptom endorsement, and diagnostic formulation to arrive at a team consensus research psychiatric diagnosis summary for all participants. This maintained categorical diagnostic fidelity between raters and minimized diagnostic drift.

For dimensional symptom inventories, such as the CDRS-R [2], clinical evaluators engaged in a measure-specific training with didactics, role plan and certification administrations observed by a study PI (VG or RHT). Prior to certification administration, clinician evaluators demonstrated at least 85% item-level agreement on co-scored taped training administrations. For longitudinal fidelity, clinical evaluators routinely co-scored recorded participant symptom inventory interviews. Additionally, to further minimize interrater variability, efforts were made to anchor participants with the same clinical evaluator longitudinally in both diagnostic interviews and symptom inventories.

To statistically evaluate inter-rater reliability for the CDRS-R [2], we computed the intraclass correlation coefficient (ICC), which has been commonly used for this scale due to its dimensional nature (i.e. continuous rather than a discrete measure) [3]. To enhance generalizability, we used a two-way random-effects model, which assumes that both participants and raters are random samples from their respective populations [4]. As detailed in **Supplementary** **Table S1** below, using this approach with three raters and six interviews, the single-rater reliability was ICC(2,1) = 0.919 (95% CI: 0.724–0.987), indicating excellent agreement for individual raters. When averaging across the three raters, reliability increased to ICC(2,3) = 0.971 (95% CI: 0.887–0.996). These values indicate a high level of consistency in coding across raters.

| **Supplementary Table S1.** CDRS-R Intraclass Correlation Coefficients | | | | | | | |
| --- | --- | --- | --- | --- | --- | --- | --- |
| Type | ICC | F | df_1_ | df_2_ | p | 95% CI Lower | 95% CI Upper |
| ICC(2,1) | 0.919 | 39.33 | 5 | 10 | 0 | 0.724 | 0.987 |
| ICC(2,3) | 0.971 | 39.33 | 5 | 10 | 0 | 0.887 | 0.996 |
| *Abbreviations*: CI = confidence interval; CDRS-R = Children's Depression Rating Scale-Revised; df = degrees of freedom; ICC = intraclass correlation coefficient; ICC(2,1) = single-rater reliability; ICC(2,3) = average reliability across three raters.  Two-way random-effects model with absolute agreement was used. | | | | | | | |

## **Suicidality Assessments**

Suicidality was assessed by both clinician interviews and self-report measures. Specifically, suicidality was evaluated as part of the semi-structured K-SADS-PL [1] interview. On the neuroimaging scan day, participants completed the Beck Scale for Suicidal Ideation (BSSI) [5]. If a participant’s BSSI score was >0, a trained clinician (psychologist or psychiatrist) conducted a safety assessment using the Columbia-Suicide Severity Rating Scale (C-SSRS) [6], along with a clinical evaluation. If the participant was deemed actively suicidal, emergency procedures would be activated to initiate admission to the nearest emergency room.

## **Practice Task Session in a Mock Scanner**

Prior to the MRI session, all participants underwent a training session of the Reward Flanker Task (RFT) in a mock scanner. During the training session, participants were familiarized with the scanning environment and learned to perform the RFT while minimizing movements. Additionally, they were informed of the performance-based bonus in order to increase motivation throughout RFT data collection.

## **Task Illustration**

**Supplementary Figure S1** depicts three possible correct outcomes following uncertain cues as part of the RFT design.

| **Supplementary Figure S1.** Example trials showing three types of correct feedback following uncertain cues in the Reward Flanker Task. |
| --- |
| 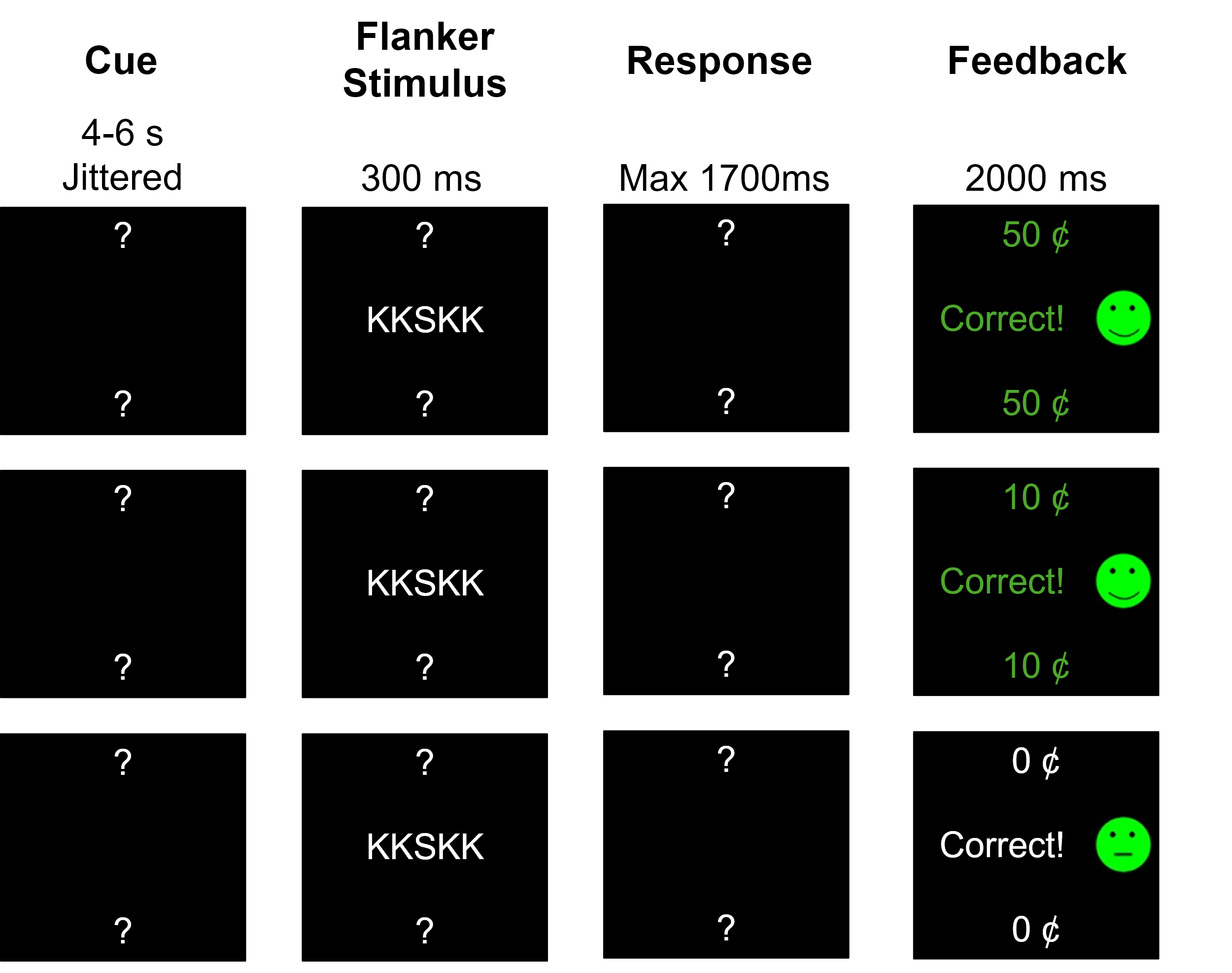 |

## **Participant Screening and Data Availability**

We enrolled 143 participants and excluded 12 who met exclusion criteria (7 with IQ < 80, 3 with neuropsychiatric disorders meeting exclusion criteria, and 2 who failed urine toxicology screenings on the scan day). 14 participants were lost to follow up and did not complete neuroimaging scans. Among 117 participants who participated in neuroimaging procedures, 10 did not have adequate MRI data acquisition due to scanner or peripheral equipment malfunction, other technical issues, or withdrawal from parts of the neuroimaging protocol leading to missing structural or functional scans. Of the 107 participants from whom RFT data were collected, data from 8 were excluded due to excessive motion in more than one RFT run (see below). Data from 15 participants were additionally excluded for reasons unrelated to motion, including scanner artifacts that negatively impacted data quality (4 participants), preprocessing issues (5 participants), and poor task performance resulting in multiple runs with empty regressors for correct outcome conditions (6 participants).

## **Data Exclusion for Excessive Motion**

Framewise head motion was rigorously examined for this study and found to be minimal. Relative displacement was calculated as the root mean squared (RMS) of 6 rigid-body movement parameter estimates generated by the HCP minimal preprocessing pipelines during realignment. Runs were excluded if >3% of frames had relative RMS displacement >1mm, while participants were excluded if more than 1 RFT run exceeded this motion limit. Data from 8 participants were excluded for having excessive motion in more than 1 RFT run. Analyses included data from 84 participants, comprising a total of 336 individual RFT runs. Of these, 8 runs (2.38%) were excluded for excessive head motion. The 328 runs included in our analyses comprised a total of 122,678 individual frames, with an average displacement (mean ± SD) of 0.115 ± 0.168mm; only 465 frames (0.38%) in the analyzed dataset exceeded 1mm of relative framewise displacement.

Notably, the exclusion rate of runs due to participant motion did not differ significantly between the clinical group (10 of 268 runs collected) and healthy control group (1 of 68 runs collected). These exclusion rates corresponded to a one-tailed *p*=0.175 (Z=0.936) using a two-proportion Z-test. Similarly, no significant correlations were detected between the RMS movement of included runs and any clinical measure: anxiety (MASC) one-tailed *p*=0.075 (*r*=0.159); depression (CDRS-R) one-tailed *p*=0.368 (*r*=0.037); anticipatory anhedonia (TEPS-AP) one-tailed *p*=0.369 (*r*=0.037); consummatory anhedonia (TEPS-CP) one-tailed *p*=0.106 (*r*=0.138); total anhedonia (TEPS) one-tailed *p*=0.182 (*r*=0.101).

# Supplementary Results

## **Details on Pubertal Status**

The inclusion criteria of Tanner stage 4 and post-menarcheal status for females were intended to address the limitation of determining pubertal status based solely on chronological age. Tanner stage was assessed using a standardized pictorial self-report scale, which was reviewed by the study physicians, including the PI (V.G.). Many potential participants were excluded and not enrolled if their Tanner stage was <4. It is important to note that depression is uncommon before puberty. As such, given that the majority of our participants were recruited from clinical settings, it is reasonable that those with depression had already reached puberty.

Across the full sample, boys (16.00 ± 2.26 years; range = 12–20) were significantly older than girls (14.87 ± 1.97 years; range = 12–20; *t* = 2.42; *p* = 0.02). In the study sample, there were 20 adolescents (16 girls, 4 boys) aged 12–13 years who had reached Tanner stage 4. When broken down by group, 16 adolescents (14 girls, 2 boys) in the mood and anxiety group and 4 adolescents (2 girls, 2 boys) in the healthy control group were 12–13 years old and had reached Tanner stage 4.

## **Correlations Between Symptoms**

| **Supplementary Table S2.** Correlations between symptoms dimensions across the full sample | | | |
| --- | --- | --- | --- |
|  | CDRS-R | MASC | TEPS |
| CDRS-R | — | — | — |
| MASC | 0.52  (*p* < 0.001) | — | — |
| TEPS | −0.47  (*p* < 0.001) | −0.05  (*p* = 0.68) | — |
| *Abbreviations*: CDRS-R = Children's Depression Rating Scale-Revised; MASC = Multidimensional Anxiety Scale for Children; TEPS = Temporal Experience of Pleasure Scale.  Correlation coefficients are reported as Pearson’s *r*. | | | |

## **Detailed Neural Responses to Reward Uncertainty during Expectancy and Attainment**

**Supplementary Figure S2** displays neural responses for the four contrasts described in the main text: *Uncertain Reward Expectancy*, *Uncertain Non-Reward Expectancy*, *Uncertain Reward Attainment*, and *Uncertain Non-Reward Attainment*. **Supplementary Tables S3** and **S4** additionally detail the significantly activated regions across these four contrasts.

| **Supplementary Figure S2.** Effect of uncertainty on expectancy and attainment phases relative to certain rewards and non-rewards: All participants. |
| --- |
| 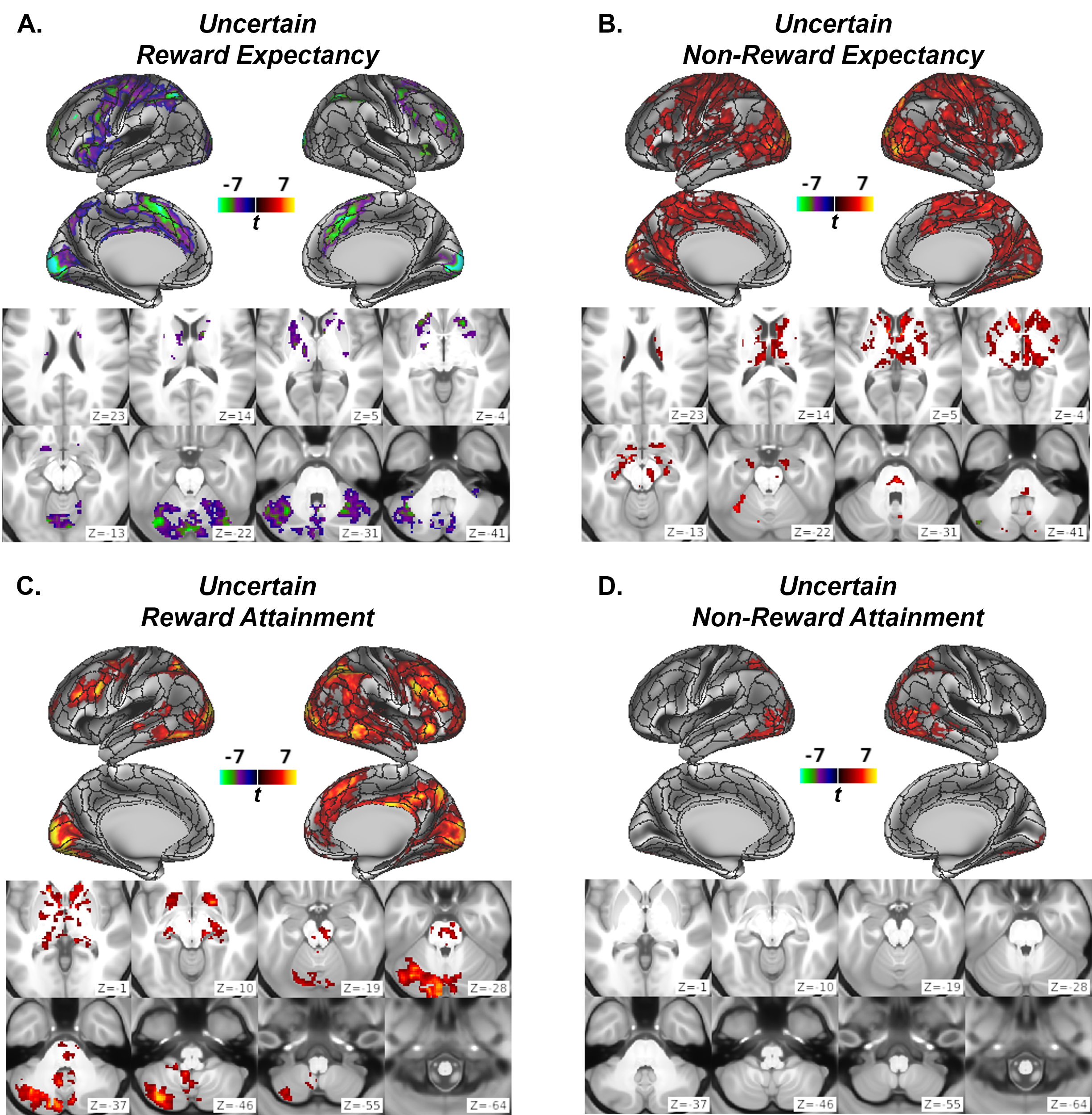 |
| Contrasts examined were **A)** *Uncertain Reward Expectancy* (uncertain (?) vs. certain reward (10¢ + 50¢) cues), **B)** *Uncertain Non-Reward Expectancy* (uncertain (?) vs. certain non-reward (0¢) cues), **C)** *Uncertain Reward Attainment* (correct reward feedback (10¢ + 50¢) following uncertain vs. certain reward cues), and **D)** *Uncertain Non-Reward Attainment* (correct non-reward feedback (0¢) following uncertain vs. certain non-reward cues). Group-level activation maps (whole sample) are displayed at the two-tailed *p_TFCE-FWE_* < 0.05 threshold. Sulcal depth from the HCP 1200-participant dataset is shown in the background. Black surface contours denote regions from the HCP MMP Atlas (Glasser et al., 2016). Subcortical results displayed in neurological convention. |

| **Supplementary Table S3.** Neural responses to *Uncertain Reward Expectancy* and *Uncertain Non-Reward Expectancy*. | | | | | |
| --- | --- | --- | --- | --- | --- |
| ***Uncertain Reward Expectancy* ^a^** | | | | | |
|  | Peak *T* | Area (mm^2^) | Cluster Overlap:  Desikan-Killiany Atlas (Desikan 2006) | Cluster Overlap: HCP MMP Atlas (Glasser 2016) | Cluster Overlap:  7-Network iFC Atlas (Yeo 2011) |
| L | –13.662 | 3146.402 | lateraloccipital (51.6%) | V1 (41.7%) | Visual (100%) |
| R | –11.758 | 2463.123 | lateraloccipital (60%) | V1 (54.2%) | Visual (100%) |
| L | –7.391 | 1109 | superiorfrontal (96.4%) | SCEF (45.6%) | Ventral Attention (61.3%) |
| R | –7.709 | 901.088 | superiorfrontal (84.4%) | a32pROI (36.7%) | Frontoparietal (50%) |
| L | –8.332 | 889.82 | superiorparietal (59.7%) | LIPd (29.3%) | Frontoparietal (49.9%) |
| R | –6.855 | 869.945 | inferiorparietal (56.3%) | IP1 (33.9%) | Dorsal Attention (54.5%) |
| L | –7.268 | 628.870 | precentral (63.1%) | FEF (61.9%) | Dorsal Attention (86.8%) |
| R | –6.835 | 603.976 | rostralmiddlefrontal (100%) | 9-46d (59.4%) | Frontoparietal (79.3%) |
| L | –6.468 | 558.25 | rostralmiddlefrontal (100%) | 9-46d (81.8%) | Frontoparietal (80.7%) |
| R | –6.819 | 522.345 | precentral (50.4%) | FEF (59.6%) | Dorsal Attention (73.6%) |
| L | –13.662 | 3146.4 | lateraloccipital (51.6%) | V1 (41.7%) | Visual (100%) |
| R | –11.758 | 2463.12 | lateraloccipital (60%) | V1 (54.2%) | Visual (100%) |
| L | –7.391 | 1109 | superiorfrontal (96.4%) | SCEF (45.6%) | Ventral Attention (61.3%) |
|  | Peak *T* | Volume (mm^3^) | Peak Coordinates (MNI X,Y,Z) | Brain Region | |
|  | –6.96 | 44176 | (30, –60, –28) | bilateral cerebellum | |
|  | –6.26 |  | (–32, –68, –20) |  |  |
|  | –6.26 |  | (0, –78, –22) |  |  |
|  | –5.06 | 4408 | (–22, –6,0) | left putamen, left palladium, left caudate | |
|  | –4.79 |  | (–22, 14, –2) |  |  |
|  | –4.75 |  | (–26, 6, 10) |  |  |
|  | –5.42 | 3512 | (32, –56, –46) | right cerebellum | |
|  | –5.29 |  | (40, –58, –54) |  |  |
|  | –4.01 |  | (38, –66, –54) |  |  |
|  | –5.61 | 2552 | (20, 14, –4) | right putamen | |
|  | –5.2 |  | (24, 6, –10) |  |  |
|  | –5.06 |  | (24, –2,10) |  |  |
|  | –4.85 | 752 | (10, 2, 8) | right caudate | |
|  | –4.44 |  | (14, 4, 14) |  |  |
|  | –3.82 |  | (16, 2, 24) |  |  |
|  | –3.78 | 584 | (–14, –6, 18) | left caudate | |
|  | –3.53 |  | (–12, 0, 14) |  |  |
|  | –3.06 |  | (–8, 8, 6) |  |  |
|  | –4.65 | 544 | (–12, –14, 6) | left thalamus | |
|  | –2.92 |  | (–8, –24, 6) |  |  |
|  | –3.61 | 376 | (18, 16, 6) | right caudate | |
|  | –3.18 |  | (18, 20, 10) |  |  |
|  | –3.51 | 176 | (–14, –16, 20) | left thalamus | |
|  | –3.37 | 144 | (30, –72, –48) | right cerebellum | |
|  | –3.21 | 72 | (–18, –20, 14) | left thalamus | |
|  | –4.11 | 72 | (14, –12, 20) | right thalamus | |
|  | –3.85 | 40 | (–8, –16, –8) | brain stem | |
| ***Uncertain Non-Reward Expectancy* ^b^** | | | | | |
|  | Peak *T* | Area (mm^2^) | Cluster Overlap:  Desikan-Killiany Atlas (Desikan 2006) | Cluster Overlap: HCP MMP Atlas (Glasser 2016) | Cluster Overlap:  7-Network iFC Atlas (Yeo 2011) |
| R | 8.211 | 3620.603 | lateraloccipital (58.3%) | V4 (11.2%) | Visual (89.9%) |
| L | 7.988 | 3516.358 | lateraloccipital (54.1%) | V4 (14%) | Visual (91.7%) |
| R | 5.995 | 1209.141 | precentral (50.8%) | 3b (28.1%) | Somatomotor (100%) |
| L | 7.081 | 1192.821 | lingual (66%) | V2 (43.4%) | Visual (100%) |
| R | 7.544 | 1107.54 | lingual (53.1%) | V4 (34.7%) | Visual (100%) |
| R | 6.125 | 767.1725 | superiorparietal (95%) | VIP (36%) | Dorsal Attention (97.7%) |
| L | 5.535 | 391.8822 | superiorparietal (100%) | VIP (50.7%) | Dorsal Attention (100%) |
| R | 5.27 | 241.4963 | precuneus (50.7%) | 5mv (58.6%) | Ventral Attention (72%) |
| R | 4.627 | 169.6892 | inferiorparietal (100%) | PGp (54.6%) | Dorsal Attention (38.6%) |
| R | 4.877 | 165.0858 | parstriangularis (100%) | IFSa (73.6%) | Frontoparietal (40.4%) |
|  | Peak *T* | Volume (mm^3^) | Peak Coordinates (MNI X,Y,Z) | Brain Region | |
|  | –4.6 | 80 | (–44, –70, –42) | left cerebellum | |
|  | –4.8 | 64 | (–42, –72, –50) | left cerebellum | |
|  | 5.48 | 33504 | (–6, 6, 2) | bilateral caudate, left accumbens, right thalamus, left amygdala | |
|  | 5.32 |  | (–6, 18, 2) |  |  |
|  | 5.29 |  | (–6, 10, –2) |  |  |
|  | 6.08 | 5792 | (–16, –48, –48) | left cerebellum | |
|  | 4.48 |  | (–22, –60, –60) |  |  |
|  | 4.16 |  | (–26, –42, –50) |  |  |
|  | 4.36 | 2192 | (12, –42, –46) | brain stem | |
|  | 4.13 |  | (2, –42, –42) |  |  |
|  | 4.12 |  | (20, –44, –46) |  |  |
|  | 4.52 | 456 | (–32, –56, –22) | left cerebellum | |
|  | 3.88 |  | (–26, –44, –24) |  |  |
|  | 4.81 | 440 | (2, –28, –32) | brain stem | |
|  | 3.03 |  | (–6, –30, –32) |  |  |
|  | 3.69 | 184 | (8, –64, –34) | right cerebellum | |
|  | 3.42 |  | (6, –60, –38) |  |  |
|  | 4.55 | 128 | (–2, –24, –46) | brain stem | |
|  | 3.69 |  | (2, –30, –46) |  |  |
|  | 4.09 | 88 | (10, –78, –50) | right cerebellum | |
|  | 3.47 | 64 | (2, –68, –46) | right cerebellum | |
|  | 3.31 | 48 | (12, –70, –44) | right cerebellum | |
| Only the largest 10 clusters and largest parcellation labels are listed here.  ^a^ 5% threshold for negative activation t-value is –4.201.  ^b^ 5% threshold for positive activation t-value is 3.885. | | | | | |

| **Supplementary Table S4.** Neural responses to *Uncertain Reward Attainment* and *Uncertain Non-Reward Attainment*. | | | | | | |
| --- | --- | --- | --- | --- | --- | --- |
| ***Uncertain Reward Attainment* ^a^** | | | | | | |
|  | Peak *T* | Area (mm^2^) | Cluster Overlap:  Desikan-Killiany Atlas (Desikan 2006) | Cluster Overlap: HCP MMP Atlas (Glasser 2016) | | Cluster Overlap:  7-Network iFC Atlas (Yeo 2011) |
| L | 9.378 | 2637.856 | lateraloccipital (68.4%) | V1 (25.3%) | | Visual (100%) |
| R | 7.841 | 2404.439 | lateraloccipital (62.1%) | V1 (36.4%) | | Visual (100%) |
| R | 8.156 | 2296.737 | inferiorparietal (62.3%) | IP1 (26.7%) | | Frontoparietal (61.2%) |
| L | 9.676 | 1702.939 | lingual (54.2%) | V2 (30.7%) | | Visual (100%) |
| R | 8.483 | 1388.473 | rostralmiddlefrontal (62.9%) | p9-46v (35.4%) | | Frontoparietal (71.1%) |
| L | 6.798 | 771.6237 | superiorparietal (51.3%) | IP1 (39.5%) | | Dorsal Attention (59.7%) |
| R | 7.245 | 752.041 | middletemporal (52.8%) | TE1p (91.3%) | | Frontoparietal (62.8%) |
| R | 6.974 | 656.5199 | lateraloccipital (49.4%) | V4 (38.5%) | | Visual (100%) |
| R | 7.449 | 505.4818 | precuneus (84.3%) | POS2 (90.4%) | | Frontoparietal (66.4%) |
| R | 6.694 | 409.3483 | isthmuscingulate (39.9%) | RSC (40.5%) | | Default (72.5%) |
| L | 9.378 | 2637.856 | lateraloccipital (68.4%) | V1 (25.3%) | | Visual (100%) |
| R | 7.841 | 2404.439 | lateraloccipital (62.1%) | V1 (36.4%) | | Visual (100%) |
| R | 8.156 | 2296.737 | inferiorparietal (62.3%) | IP1 (26.7%) | | Frontoparietal (61.2%) |
|  | Peak *T* | Volume (mm^3^) | Peak Coordinates (MNI X,Y,Z) | Brain Region | | |
|  | 7.05 | 32536 | (20, 10, –6) | bilateral thalamus, putamen, caudate and accumbens | | |
|  | 6.06 |  | (8, –14, 8) |  |  |  |
|  | 5.82 |  | (–6, 2, 6) |  |  |  |
|  | 7.48 | 30136 | (–12, –76, –36) | left cerebellum | | |
|  | 7.34 |  | (–16, –80, –34) |  |  |  |
|  | 6.77 |  | (–8, –86, –24) |  |  |  |
|  | 4.2 | 200 | (–12, –18, –26) | brain stem | | |
|  | 4.1 | 168 | (14, –22, –36) | brain stem | | |
|  | 3.06 |  | (16, –20, –28) |  |  |  |
|  | 3.21 | 56 | (0, –36, –30) | brain stem | | |
|  | 3.79 | 48 | (0, –44, –20) | brain stem | | |
|  | 3.48 | 32 | (–4, –28, –18) | brain stem | | |
|  | 3.03 | 16 | (–6, –16, –24) | brain stem | | |
| ***Uncertain Non-Reward Attainment*** | | | | | | |
|  | Peak *T* | Area (mm^2^) | Cluster Overlap:  Desikan-Killiany Atlas (Desikan 2006) | Cluster Overlap: HCP MMP Atlas (Glasser 2016) | | Cluster Overlap:  7-Network iFC Atlas (Yeo 2011) |
| R | 5.524 | 10584.06 | lateraloccipital (28.6%) | V4 (8.8%) | Visual (48.6%) | |
| L | 5.28 | 5463.737 | lateraloccipital (34.0%) | PH (13.1%) | Visual (51.5%) | |
| Only the largest 10 clusters and largest parcellation labels are listed here.  ^a^ 5% threshold for positive activation t-value is 4.861. | | | | | | |

# Supplementary Discussion

## **Noise Removal Considerations**

With respect to noise removal, a recent analysis by Hoeppii and colleagues [7] found that ICA-FIX yielded the best trade-off between signal preservation and noise reduction for task fMRI data compared to ICA-AROMA, PCA-based anatomical or temporal CompCor regression, or standard preprocessing without regression-based denoising. Studies examining the benefits of motion censoring have meanwhile been largely equivocal, both in the context of task-based fMRI designs [8] and accelerated fMRI data collected by the HCP with high spatiotemporal resolution and processed using very similar methodology to our own [9]. Given the low levels of movement in our dataset as well as the high quality of the acquisitions and preprocessing, including manual review of all ICA components retained by FIX, no additional censoring was performed. We are confident that this approach sufficiently addresses potential motion-related artifacts.

## **Response Interval Calibration**

Our group initially described development of the Reward Flanker Task (RFT) in a pilot study of 22 adolescents [10]. We used an individualized response window of 1.5 × the participant’s mean response time during the final segment of pre-scan task training, which matched the length and timing parameters of the in-scanner RFT runs. Due to timing constraints during the scan (e.g. fixed TR), the response window was capped at a maximum of 1700ms following the end of the 300ms flanker stimulus. In our pilot study, we found that this window allowed participants to achieve a mean accuracy of 85.0% ± 15.9%. The current study as well as a related prior publication [11] used the same response window calculation during the RFT in expanded sample of 84 adolescents and achieved similar accuracy of 86.6% ± 9.1%. The RFT itself was developed by one of our coauthors, Dr. Emily Stern, building on her earlier Incentive Flanker Task [12], which ultimately traced to the widely used Monetary Incentive Delay and Flanker tasks, as described in our **Methods** section. While the earlier Incentive Flanker Task used a variable response window of 0.8-1.5 × the participant’s mean response time during a practice session, the window length was explicitly selected to achieve an accuracy of ~85%. As such, scaling individuals’ mean response times from practice RFT sessions by a fixed 1.5 × factor consistently achieves the target accuracy for our study population. That this scaling factor is at the maximum of the range established for the predecessor Incentive Flanker Task likely reflects the greater complexity and cognitive burden of the RFT, which includes additional uncertain cues not present in the Incentive Flanker Task.

**Supplementary References**

1 Kaufman J, Birmaher B, Brent D, Rao U, Flynn C, Moreci P, et al. Schedule for Affective Disorders and Schizophrenia for School-Age Children-Present and Lifetime Version (K-SADS-PL): initial reliability and validity data. J Am Acad Child Adolesc Psychiatry. 1997;36(7):980-8.

2 Poznanski EO, Mokros HB. Children's Depression Rating Scale, Revised (CDRS-R) Manual*.* Western Psychological Services: Los Angeles; 1996.

3 Hytman L, Mansueto S, Chan JI, Kumar R, Nguyen ATP, Wang W, et al. Interrater Reliability and Measurement Error of the Children’s Depression Rating Scale–Revised in Adolescents. JAACAP Open. 2025.

4 Shrout PE, Fleiss JL. Intraclass correlations: uses in assessing rater reliability. Psychol Bull. 1979;86(2):420-8.

5 Beck AT, Kovacs M, Weissman A. Assessment of suicidal intention: the Scale for Suicide Ideation. J Consult Clin Psychol. 1979;47(2):343-52.

6 Posner K, Brown GK, Stanley B, Brent DA, Yershova KV, Oquendo MA, et al. The Columbia-Suicide Severity Rating Scale: initial validity and internal consistency findings from three multisite studies with adolescents and adults. Am J Psychiatry. 2011;168(12):1266-77.

7 Hoeppli ME, Garenfeld MA, Mortensen CK, Nahman-Averbuch H, King CD, Coghill RC. Denoising task-related fMRI: Balancing noise reduction against signal loss. Human Brain Mapping. 2023;44(17):5523-46.

8 Jones MS, Zhu Z, Bajracharya A, Luor A, Peelle JE. A Multi-Dataset Evaluation of Frame Censoring for Motion Correction in Task-Based fMRI. Apert Neuro. 2022;2:1-25.

9 Phạm DĐ, McDonald DJ, Ding L, Nebel MB, Mejia AF. Less is more: balancing noise reduction and data retention in fMRI with data-driven scrubbing. NeuroImage. 2023;270:119972.

10 Bradley KAL, Case JAC, Freed RD, Stern ER, Gabbay V. Neural correlates of RDoC reward constructs in adolescents with diverse psychiatric symptoms: A Reward Flanker Task pilot study. Journal of Affective Disorders. 2017;216:36-45.

11 Liu Q, Ely BA, Stern ER, Xu J, Kim J-w, Pick DG, et al. Neural function underlying reward expectancy and attainment in adolescents with diverse psychiatric symptoms. NeuroImage: Clinical. 2022;36:103258.

12 Stern ER, Welsh RC, Fitzgerald KD, Gehring WJ, Lister JJ, Himle JA, et al. Hyperactive error responses and altered connectivity in ventromedial and frontoinsular cortices in obsessive-compulsive disorder. Biol Psychiatry. 2011;69(6):583-91.
